# Spatial statistical and environmental correlation analyses on vector density, vector infection index and Japanese encephalitis cases at the village and pigsty levels in Liyi County, Shanxi Province, China

**DOI:** 10.1186/s13071-022-05305-8

**Published:** 2022-05-19

**Authors:** Mei-De Liu, Chun-Xiao Li, Jing-Xia Cheng, Tong-Yan Zhao

**Affiliations:** 1 Academy of Military Medical Sciences State Key Laboratory of Pathogen and Biosecurity, Beijing, 100071 People’s Republic of China; 2 Shanxi Centers for Disease Control and Prevention, Taiyuan, 030012 People’s Republic of China

**Keywords:** Mosquito vector, Japanese encephalitis, Environment, Pigsty, Spatial analysis

## Abstract

**Background:**

In the eco-epidemiological context of Japanese encephalitis (JE), geo-environmental features influence the spatial spread of the vector (*Culex tritaeniorhynchus*, Giles 1901) density, vector infection, and JE cases.

**Methods:**

In Liyi County, Shanxi Province, China, the spatial autocorrelation of mosquito vector density, vector infection indices, and JE cases were investigated at the pigsty and village scales. The map and Enhanced Thematic Mapper (ETM) remote sensing databases on township JE cases and geo-environmental features were combined in a Geographic Information System (GIS), and the connections among these variables were analyzed with regression and spatial analyses.

**Results:**

At the pigsty level, the vector density but not the infection index of the vector was spatially autocorrelated. For the pigsty vector density, the cotton field area was positively related, whereas the road length and the distance between pigsties and gullies were negatively related. In addition, the vector infection index was correlated with the pigsty vector density (PVD) and the number of pigs. At the village level, the vector density, vector infection index, and number of JE cases were not spatially autocorrelated. In the study area, the geo-environmental features, vector density, vector infection index, and JE case number comprised the Geo-Environment-Vector-JE (GEVJ) intercorrelation net system. In this system, pig abundance and cotton area were positive factors influencing the vector density first. Second, the infection index was primarily influenced by the vector density. Lastly, the JE case number was determined by the vector infection index and the wheat area.

**Conclusions:**

This study provided quantitative associations among geo-environmental features, vectors, and the incidence of JE in study sties, one typical northern Chinese JE epidemiological area without rice cultivation. The results highlighted the importance of using a diverse range of environmental management methods to control mosquito disease vectors and provided useful information for improving the control of vector mosquitoes and reducing the incidence of JE in the northern Chinese agricultural context.

**Graphical Abstract:**

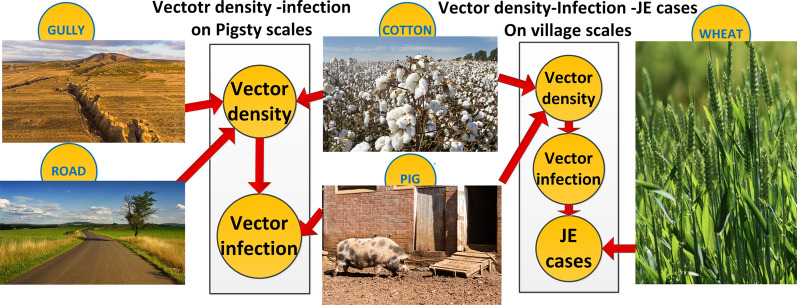

**Supplementary Information:**

The online version contains supplementary material available at 10.1186/s13071-022-05305-8.

## Background

Japanese encephalitis (JE) is a major mosquito-borne disease transmitted among humans, pigs, and birds by insect bites [1]. *Culex tritaeniorhynchus* (Giles, 1901) is considered a key vector of JE in northern China [2], and its larvae can be found in rice paddies, ponds, channels, and other sites with clear water as well as large water bodies [3]. Without successful antiviral treatment for viral infection, JE fatality rate can reach 30%, and half of the survivors have to face lifetime neuropsychiatric damage [4, 5]. In China, JE is currently the most broadly distributed and dangerous mosquito-borne disease [6, 7]. Particularly in the study area, which is located in northern China, JE is considered the most important mosquito-borne disease and poses a significant threat to public health [8]. Thanks to safe and effective vaccines specific for JE, excellent childhood vaccination programs can reduce the infection rate and financial burden in JE epidemic countries.

Although immunization has previously kept the incidence of JE in this region comparatively low [9], the incidence of the disease has increased [10]. Particularly in 2006, an outbreak of JE in Liyi County, Shanxi Province, attracted widespread national interest because this disease was thought to have been effectively controlled in the region [10]. Previous research has shown that the spatial distribution of mosquito vectors and the JE virus are associated [11, 12]. Their spatial distribution appears to be determined by various geo-environmental features, mosquito density, and infection rate [13–15]. Thus, the outbreak of JE in Liyi County, Shanxi Province, in 2016 led us to wonder about the relationship between geo-environmental features and JE cases as well as their vectors and which factors could contribute to the relapse of JE.

The 3S technology, including geographic information systems (GIS), remote sensing (RS), and global positioning systems (GPS), is widely used in vector-borne disease epidemiology research [16, 17]. With the advantage of being a large-scale, real-time, and accurate 3S technology, researchers can discover the connection between vector-borne disease (JEV, for example) and geo-environmental features [18]. In this study, GPS was used to locate the pigsties, RS was used to identify and quantify the geo-environmental features, and GIS was used to construct the spatial datasets of the vector and geo-environmental features. Consequently, we attempted to determine the spatial distribution characteristics and infection rate of *Cx. tritaeniorhynchus* in Liyi County, Shanxi Province, China, and the relationship between these and local geo-environmental features by combining spatial analysis, 3S methods, and regression.

In China, the JE cases were linked to the geo-environmental features [19, 20], and the vector distribution map coincided with areas of high JE incidence [21], which emphasized the association of the geo-environmental features with JE vectors as well as JE cases. In this study, we present the results of the analysis on the spatial dependence among the geo-environmental features, *Cx. tritaeniorhynchus* density, and JE infection rate in villages in northern China. From an epidemiological standpoint, the results in the present paper could provide an environmental-ecological explanation for the outbreak of JE cases in Shanxi Province. Furthermore, this study provides clues for reducing the incidence of JE in this region.

## Methods

### Study site

**Fig. 1:**
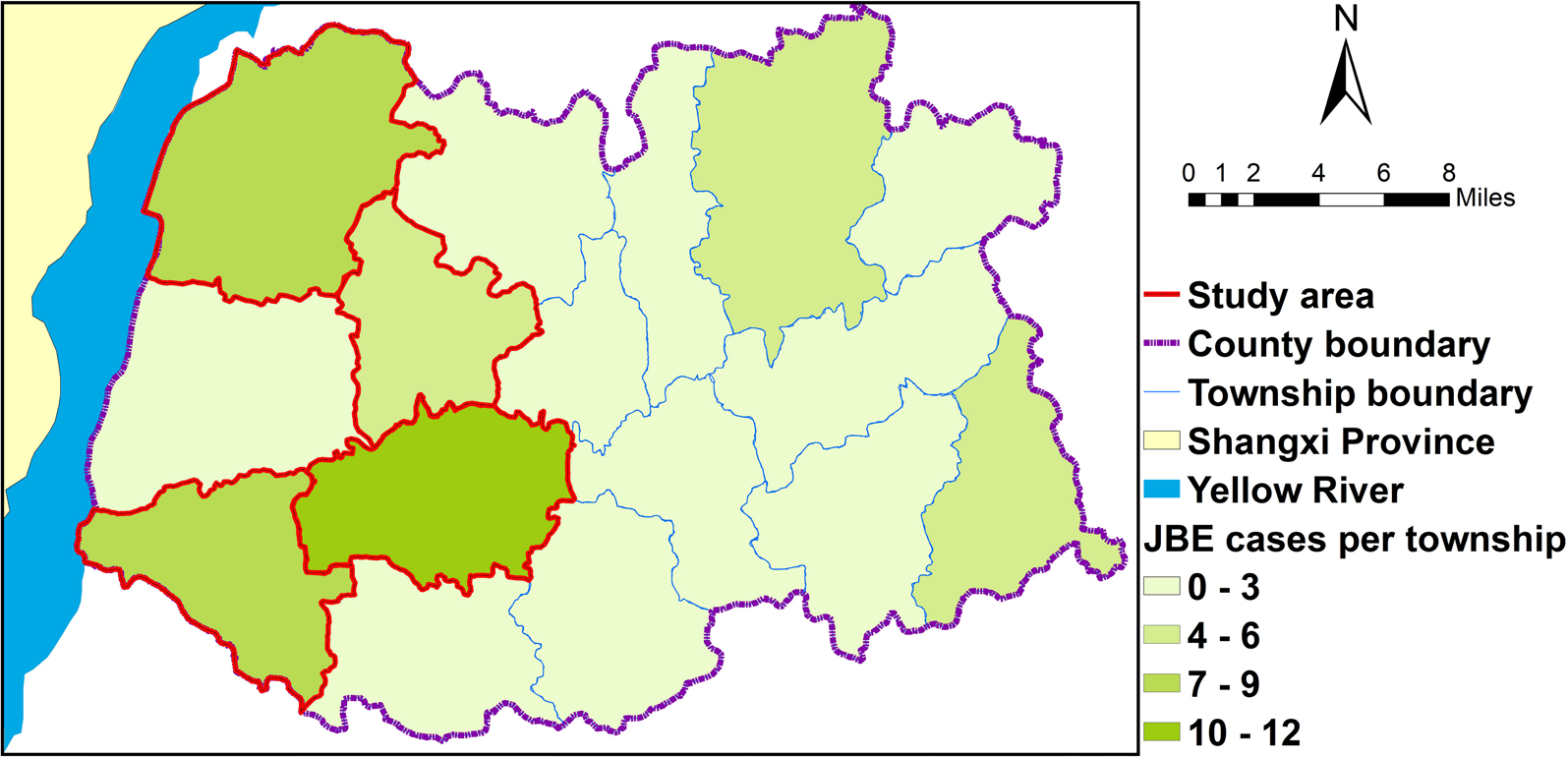
Frequency of Japanese encephalitis cases at the district level in LiYi County, Shanxi Province, China

Liyi County is part of Yuncheng prefecture, which is located in southern Shanxi Province. The major subsistence crops in this region are corn and wheat, and the major commercial crops are vegetables, orchards, and cotton. In 2006, almost half the JE cases in Liyi County were in Yuncheng prefecture [22]. We selected four districts in Liyi County as study sites, and 54% (33/61) of all the JE cases recorded in Liyi County between 2005 and 2009 were in these districts (Fig. 1).

### Mosquito trapping in pigsties

**Fig. 2:**
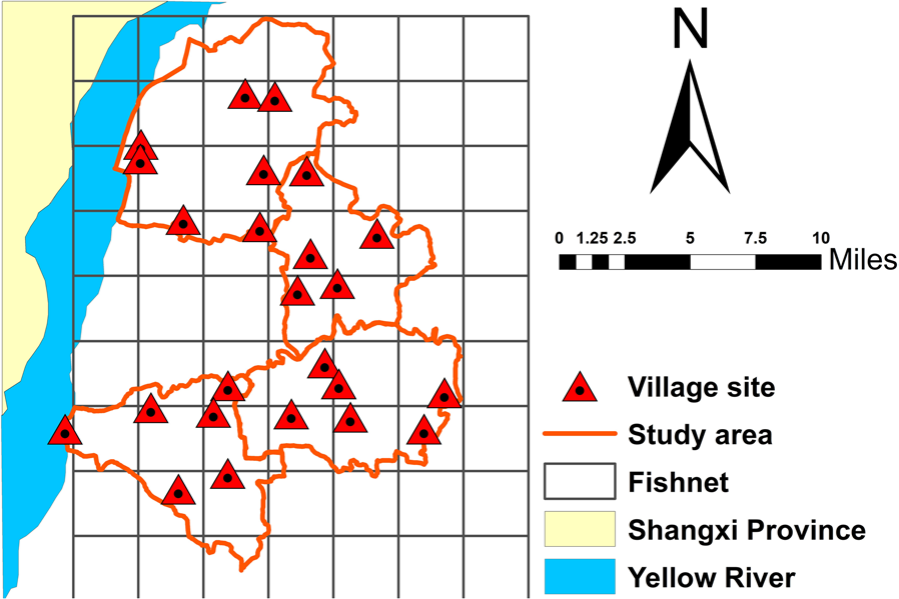
Map of Liyi County, Shanxi, China showing study area and the locations of villages chosen as study-sites

The four districts selected as study area were first further subdivided into 24 × 4 km^2^ subareas (Fig. 2). In each subarea, one village was then randomly selected as a study site, giving a total of 24 study sites. Additionally, three or four pigsties in each village were selected as mosquito surveillance positions. Finally, we had 93 pigsties for vector investigation.

In Liyi County, the *Cx. tritaeniorhynchus* population density peaks in June, July, and August. Therefore, mosquitoes were trapped continuously over a 7-day period in the middle of months with peak vector abundance (June–August) from 2010 to 2011. Mosquitoes were trapped with light traps (LTS-M02B Chinese CDC light 220 V/50HZ 24 W, Wuhan Lucky Star Environmental Protection Technology Co., Ltd., Hubei, China) in 93 pigsties distributed throughout the 24 village study sites. Inside each pigsty, one light trap was hung 1.5-m height from the floor, and the light was turned on at 7:00 p.m. and off at 7:00 a.m. daily. On bad weather days, trapping was postponed one more day. Every day, the mosquito samples were collected and transported to the laboratory for later identification, and the trapped female mosquitoes were identified by morphology and counted.

### Calculation of mosquito density and infection index

The mosquito density (N) was calculated from the light trap density as follows:

$$ {\text{N}} = {\text{MN}}/{\text{LN}} $$

where MN is the number of trapped mosquitoes, and LN is the number of light traps. The vector densities of the pigsty (PVD) and the village (VVD) were determined at the pigsty and village scales, respectively.

Reverse transcription polymerase chain reactions (RT-PCRs) were performed on mosquito batches to determine the proportion of mosquitoes infected with the JE virus. The infection index was calculated according to the method described by [23]

$$ {\text{VI}} = \sum\limits_{{i = {\text{species}}}} {\overline{{N_i}}} \hat{P}_i $$

where $\overline{\text{N}_\text{i}}$ is the average mosquito density and $\hat{P}_{\text{i}}$ is the estimated infection rate of the mosquito population [24]. The pigsty vector infection indices of the (PVII) and the village (VVII) were also determined at the pigsty and village scale, respectively.

### JE epidemic in village sites

Baseline data on the incidence of JE in the 24 study villages over the 5-year period from 2005 to 2009 were acquired from the Liyi County Centers for Disease Control (CDC). These data included detailed information on each JE case, such as the name, sex, age, village, and diagnostic procedure, and they were incorporated into the GIS spatial vector database by attaching them to each village’s location. The JE case number of villages (VJC) was deduced from the JE epidemic datasets.

### GPS, RS, and GIS analysis of the study site image dataset

The location of each sampled pigsty was determined by GPS (Trimble GeoXT 2005, Sunnyvale, CA) before trapping. The Enhanced Thematic Mapper (ETM) satellite images (LandSat ETM 2009-9-27 with 15-m resolution) covering Liyi County were ordered from EarthView Image Inc. (Beijing, China), which also took responsibility for correcting the scan line error on the ETM images that minimized the error for remote sensing as much as possible. The numbers of pigsties and vegetation, such as wheat, orchards, cotton, corn, and vegetables, were regarded as geo-environmental features that could potentially affect the mosquito density. First, we investigated the environment around each pigsty and village and recorded the vegetation type, position, and living stage for the field period. Then, the ETM images were classified with ENVI 4.7 (ESRI, Redlands, CA) using unsupervised classification method-ISODATA (number of classes: maximum 20, minimum 10; maximum iteration: 5; change threshold%: 5.00).

Some geographic factors, such as rivers, roads, and gullies, could also potentially affect mosquito abundance in pigsties. The vectorization of these factors was performed in ArcGIS10.0 (ESRI, Redlands, CA) based on the Liyi county road transport map.

### Extraction of spatial factors

On the ETM image of each study site, a 1-km circular buffer around each pigsty and village was drawn in ArcGIS using the Buffer tool in the Arc Toolbox. The vegetation and geographic factors inside each buffer circle (around the pigsty and village) were then generated through spatial extraction with the HawthsTools (v3.x) extension (Beyer, H L, 2004). With HawthsTools, the resulting polygonal factor area was then extracted with the “Polygon in Polygon Analysis” tool, while the line cause length was finished using the “Sum Line Lengths in Polygons” tool. The distances from the pigsties to the land cover and geographic factors were measured in ArcGIS with the Near tool in Arc Toolbox.

### Geo-environmental features for analysis

**Fig. 3:**
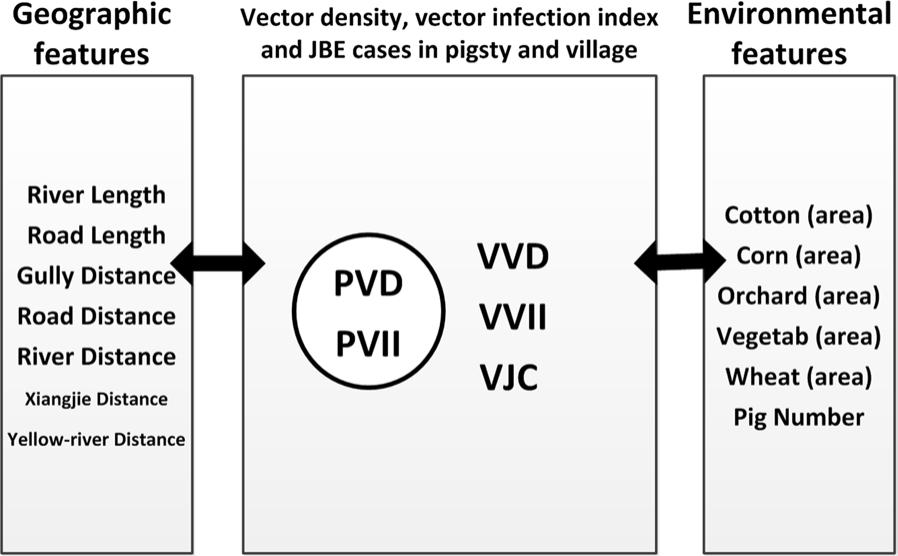
Geo-environmental features potentially correlated with the vector density, vector infection index, and Japanese encephalitis cases at the scales of pigsty and village. *PVD* pigsty vector density; *PVII* pigsty vector infection index; *VVD* village vector density; *VVII* village vector infection index; *VJC* village Japanese encephalitis case number

The geo-environmental features that were potentially correlated with the abundance of vectors, the vector infection index, and the village Japanese encephalitis case number in the study area are pictured in Fig. 3. The major geo-environmental features included the cotton area [area-area of the features within a 1 km buffer around the village (pigsties)], corn area, orchard area, vegetable area, and wheat area. The geographic features were the river length [length-length of features within a 1 km buffer around the villages (pigsties)], road length, gully distance [distance-distance between villages (pigsties) and features], road distance, river distance, Xiangjie (division line of districts) distance, and Yellow River distance. Lastly, the pig number (growing pig number) was also counted because of its important host role in JE transmission. We performed the study at the pigsty and village scales, considering the spatial scale influence of the geo-environmental features on the local vector and the epidemiology of JE (see Additional file 1: Table S1).

### Statistical analysis scales and significance threshold

The spatial autocorrelation and regression analyses were conducted at the pigsty and village scales, respectively. All statistical analyses were performed using R version 3.4.4 (R Core Team, 2018). Statistical significance was set at *P* < 0.05. The Bonferroni correction was applied when required in the evaluation of multiple comparison results.

### Distribution and correlative test of the variable

First, the variables of the geo-environmental features were normalized using BOX-COX transformation in R. Then, the one-sample Kolmogorov-Smirnov test was performed to detect the statistical distribution of the vector variables (PVD, PVII, VVD and VVII) and the JE case number (VJC); if they obeyed the normalization distribution, the Pearson correlative analysis and linear regression model were used; otherwise, the Spearman rank correlative analysis and generalized linear regression model were employed.

### Spatial autocorrelation detection

The univariate Moran’s test (Queen Contiguity Weight was used) in GeoDa (095i) (GeoDa Center for Geospatial Analysis and Computation, Tempe, AZ) was first used to detect the spatial autocorrelation of PVD, PVII, VVD, VVII, and VJC by Moran index calculation. Second, the inference for the Moran index was based on a random permutation procedure in GeoDa with 999 rounds. If the *P*-value of the permutations was < 5%, then the spatial autocorrelation was statistically significant; otherwise, the spatial distribution was random. If significant spatial autocorrelation was detected, the relationship between the dependent variable and geo-environmental features (covariates) was analyzed using the spatial regression package within GeoDa. If not, this type of analysis was conducted using a linear model or generalized linear model (Poisson GLM) in the R system [25], according to the statistical distribution test (Kolmogorov-Smirnov test).

### Spatial regression

If spatial autocorrelation was detected for the vector variables and JE case number, the ordinary least squares (OLS) model, spatial lag model (SLM), and spatial error model (SEM) were applied simultaneously, and the *P*-values of the OLS (*F* test), spatial lag model (Lagrange multiplier test), and spatial error model (Lagrange multiplier test) were compared to confirm whether spatial regression is necessary. Furthermore, a comparison of the Akaike information criterion (AIC) value between the spatial lag model and spatial error model was used to diagnose which spatial model would be more fitted for the spatial regression analysis.

Once the spatial regression model was determined, the independent variables correlated to the dependent variable were included in the spatial regression model with diversity variable combinations. The model with the lowest AIC value was considered the most appropriate model, and an AIC value difference of < 2 between the two models suggested that there was no significant difference in explanatory power of two models.

### Linear and generalized linear model regression

Using the linear regression model [with lm () function in R system], the correlated features of the dependent variables were included in the model in a stepwise way, which could exclude possible collinearity in the dependent variables and result in the most explanatory model.

In the generalized linear model regression [with glm () function in R system], the independent variables correlated to the dependent variable were included in the regression model with diversity variable combinations, excluding possible collinearity among the dependent variables. The model with the lowest AIC value was considered the most appropriate model, and the AIC value difference was < 2 between the two models, suggesting that there was no significant difference in their explanatory power.

## Results

### Surveillance data

In 93 pigsties of 24 villages, 13,492 mosquitoes were captured by light traps, including *Cx. tritaeniorhynchus*, *Cx. pipens pallens* (Coquillett, 1898), *Anopheles sinensis* (Wiedemann, 1928), *Armigerini subalbatus* (Coquillett, 1898), *Aedes dorsalis* (Meigen, 1830), and *Ae. Albopictus* (Skuse, 1894). The number and percentage of these species were ranked as *Cx. tritaeniorhynchus* (6462, 47.90%), *Cx. pipens pallens* (6252, 46.34%), *An. sinensis* (386, 2.86%), *Ar. subalbatus* (225, 1.67%), *Ae. dorsalis* (120, 0.98%), and *Ae. Albopictus* (47, 0.53%).

### Regression analysis of PVD and selected geo-environmental features

As shown by the regression results in Geoda (see Additional file 1: Table S2), the *P*-value of OLS was > 0.05, and the *P*-values of both the Lagrange multiplier (lag) test and Lagrange multiplier (error) test were < 0.05. In addition, the AIC value of the robust LM (error) test was significantly smaller than that of the robust LM (lag) test. Finally, the Moran index and its random permutation test showed that there was significant spatial autocorrelation in the PVD (see Additional file 1: Table S3, Fig. S1). Consequently, the spatial error model provided a better approximation of the PVD.

As shown by spatial error regression model results, the cotton area (*P* = 0.002), gully distance (*P* = 0.027), and road length (*P* = 0.027) were significant coefficients with the PVD in the context of the spatial error regression model, without intercorrelation among them. Thus, these three factors were introduced into the spatial error regression model in a stepwise fashion, which resulted in seven models (see Additional file 1: Model S0.1–Model S0.7). Following a spatial error regression analysis on these seven models and comparing the AIC value, the AIC values of models 0.3, 0.5, and 0.7 were significantly smaller than those of model 0.1, 0.2, 0.4, and 0.6.

Among models 0.3, 0.5, and 0.7, model 0.7 provided a full explanation of the link between the geo-environmental features and the PVD, and the test of heteroskedasticity (Breusch-Pagan test, *P* = 0.54) and spatial error dependence (likelihood ratio test of *P* = 0.04) also suggested that model 0.7 performed better than model 0.3 and 0.5. According to model 0.7, the cotton area correlated positively with the PVD (coefficient = 1.45 e−004) with 95% confidence intervals (1.36 e−004, 1.54 e−004) and the gully distance (coefficient = − 0.014) with 95% confidence intervals (− 0.0161, − 0.0136); road length (coefficient = − 0.041) with 95% confidence intervals (− 0.044, − 0.036) correlated negatively to the PVD at a significant level.

### Generalized linear model (GLM) regression analysis of PVII

The PVII followed the Poisson distribution and displayed non-significant spatial autocorrelation (see Additional file 1: Table S3, Fig. S2). So, the PVD (*P* = 0.007) and pig number (*P* = 0.043) were selected by Spearman rank analysis and introduced into the Poisson regression model in a stepwise way, which resulted in three models (see Additional file 1: Model S1.1–Model S1.3). Following a GLM regression analysis on these three models and comparing the AIC value, model 1.3 had a significantly smaller value than models 1.1 and 1.2. Subsequently, a Poisson GLM was conducted on model 1.3, and its results are listed in Table 1. Based on the GLM regression results, model 1.3 fit our data well and was significantly different from the model with only one intercept (likelihood ratio chi-square = 20.213, *P* < 0.001), and the PVD (*P* < 0.001) and pig number (*P* < 0.001) were all significantly and positively correlated with the PVII.

**Table 1:** Results of Poisson regression in model 1.3

Factor	Coefficient	95% confidence intervals		Hypothesis test		Model omnibus test	
		Lower limit	Upper limit	Wald chi-square	Sig	Likelihood ratio chi-square	Sig
Intercept	− 1.937	− 3.197	− 0.677	9.077	0.003	20.213	< 0.001
PVD	0.01	0.006	0.014	22.628	< 0.001		
Pig number	0.015	0.007	0.022	12.916	< 0.001		

### Linear regression analysis of the vector density of villages (VVD) and geo-environmental features

At the village level, the village vector density (VVD) followed the normal distribution and displayed non-significant spatial autocorrelation (see Additional file 1: Table S3, Fig. S3), and the cotton area (P = 0.019) and pig number (*P* = 0.024) were significantly associated with VVD. Consequently, the cotton area and pig number were incorporated into the linear regression analysis of the relationship between the vector density and geo-environmental features (Table 2). The analysis of variance (ANOVA) results for the linear regression model (Table 2) indicated an F value of 6.06 (*P* = 0.009), which indicated that the linear regression model significantly accounts for the linear relationship from the actual trap data. In addition, the model coefficients (Table 2) showed a significant, positive relation among the cotton area (*P* = 0.036), pig number (*P* = 0.046), and VVD.

**Table 2:** Linear regression model analysis on the vector density and geo-environmental features

Factor	Pearson correlation		Coefficient analysis			t	Sig	ANOVA model	
	Correlation	Sig	Coefficients	95% confidence intervals				F	Sig
				Lower limit	Upper limit				
(Constant)	Na	Na	1.392	0.612	2.172	3.721	0.001	6.06	0.009
Pig number	0.469	0.024	0.013	0.00023	0.025	2.13	0.046		
Cotton area	0.486	0.019	4.35e−006	0.00 e−006	8.0e−006	2.25	0.036		

### Generalized linear regression on the village vector infection index (VVII) and geo-environmental features

The VVII obeyed the Poisson distribution and displayed non-significant spatial autocorrelation (see Additional file 1: Table S3, Fig. S4). Thus, the VVD (*P* < 0.001), river length (*P* = 0.021), and pig number (*P* = 0.031) were selected and involved in the GLM regression model in a stepwise way, which resulted in five models (see Additional file 1: Model S2.1–Model S2.5). Note that the pig number and VVD could not be input into the model at the same time because of the significant intercorrelation between the VVD and pig number.

Following the GLM regression analysis on these five models and comparing the AIC value, model 2.1 had a significantly lower AIC than model 2.2, 2.3, 2.4, and 2.5. Subsequently, the results of Poisson GLM on model 2.1 are listed in Table 3. In Table 3, model 2.1 fitted our data well and significantly different from the model with only one intercept (likelihood ratio chi-square = 11.13, *P* < 0.001), and the VVD (*P* < 0.001) was significantly and positively related to the VVII.

**Table 3:** Results of Poisson regression in model 2.1

Factor	Coefficient	95% confidence intervals		Hypothesis test		Model omnibus test	
		Lower limit	Upper limit	Wald chi-square	Sig	Likelihood ratio chi-Square	Sig
Intercept	− 6.36	− 8.583	− 4.137	31.439	< 0.001	11.13	0.001
VVD	1.448	0.928	1.968	29.79	< 0.001		

Because of the lower *P* value of Moran’s index test of VVII (pseudo-*P*-value = 0.084), we also performed the diagnostics for spatial dependence test on spatial lag regression and spatial error regression model on VVII. Both the probability of LM [Lagrange multiplier (lag), *P* = 0.774] test and LM [Lagrange multiplier (error), *P* = 0.736] were significantly bigger than 0.05, which confirmed that the spatial regression was not appropriate for VVII.

### Generalized linear regression on village JE cases and geo-environmental features

The VJC obeyed Poisson distribution and displayed non-significant spatial autocorrelation (see Additional file 1: Table S3, Fig. S5). Thus, the VVII (*P* = 0.026), wheat area (*P* = 0.031), Xiangjie distance (*P* = 0.027), and pig number (*P* = 0.024) were introduced into the Poisson regression model in a stepwise way, which resulted in four models (see Additional file 1: Model S3.1–Model S3.4).

Following a GLM regression analysis on these five models and comparing the AIC value, model 3.2 had a significantly lower AIC value than models 3.1, 3.3, and 3.4. Subsequently, the results of Poisson GLM on model 3.2 are listed in Table 4. In Table 4, model 3.2 fit our data well and was significantly different from the model with only one intercept (likelihood ratio chi-square = 9.429, *P* < 0.001), and the VVD (*P* < 0.001) and wheat area (*P* < 0.001) were positively and significantly associated with the VJC.

**Table 4:** Results of Poisson regression in model 3.2

Factor	Coefficient	95% confidence intervals		Hypothesis test		Model omnibus test	
		Lower limit	Upper limit	Wald chi-square	Sig	Likelihood ratio chi-square	Sig
Intercept	− 1.170	− 1.817	− 0.523	12.571	< 0.001	9.429	0.009
VVII	1.345	0.633	2.056	13.724	< 0.001		
Wheat area	0.6	0.318	0.881	17.436	< 0.001		

### Correlation net among geo-environmental features, vector density, vector infection index, and JE case number at the pigsty and village levels

**Fig. 4:**
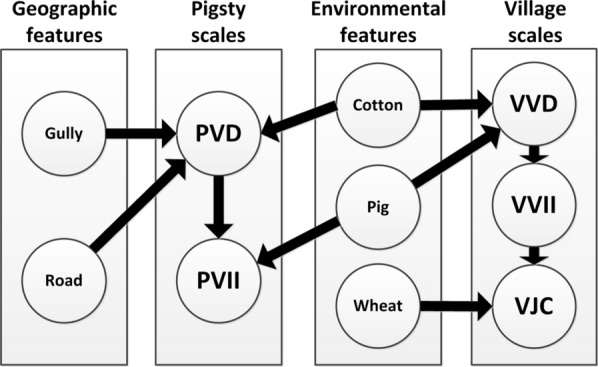
Correlation network diagram on geo-environmental features, vector density, vector infection index, and Japanese encephalitis case number at the scales of pigsty and village. *PVD* pigsty vector density; *PVII* pigsty vector infection index; *VVD* village vector density; *VVII* village vector infection index; *VJC* village Japanese encephalitis case number

In the study area, there was a net correlation among the geo-environmental features, vector density, vector infection index, and JE case number at the pigsty as well as the village level. At the pigsty scale, the geographic features (gully distance and road length) were first related to the PVD and thus indirectly related to PVII (gully distance and road length were discarded because of their correlation with the PVD); moreover, the cotton area was related to the PVD and then the PVII (cotton area was discarded because of its correlation with the PVD); in addition, the pig number was directly related to the PVII. On the village scale, the geo-environmental features (cotton area and pig number) were first related to the VVD, then to the VVII, and finally to the VJC (the cotton area and pig number were discarded because of their correlation with the VVD); in addition, the wheat area was directly related to the VJC. Overall, there was a JE epidemiological interrelation net in the study area, as shown in Fig. 4.

## Discussion

In northern China, the present study uncovered that arid crop growth could also affect the mosquito vector population and JE cases. Generally, rice cultivation is considered a major factor that influences the spread of JE in southern China [26, 27]. In northern China, arid crops are predominantly grown because of the shortage of water particularly at our study site. The primary crops include cotton, wheat, corn, orchard, and some vegetables. Because of water shortages, this type of agricultural pattern provides few breeding sites for mosquitoes [28], and there is no previous study that mentions the association among arid crop growth, JE cases, and vector density. Our results were the first to our knowledge to indicate that the JE vector density at the pigsty and village levels was positively correlated with the cotton farming area. In our study area, cotton farmers usually water their cotton fields by periodic flooding, which creates abundant favorable breeding sites for JE vector mosquitoes, the larvae of which are often found in cotton fields [29]. Thus, cotton irrigation methods may explain the positive association between cotton farming and vector density. However, the positive connection between the wheat growing area and the JE cases at the village scale was difficult to explain here. We suspected that it could be an artifact of a positive relation between wheat production and pig farming, and further investigation is necessary. Although rice cultivation is considered a major factor contributing to epidemiology of JE in southern China [26, 27], the relationship between geo-environmental features and JE in northern China has not previously been determined. Therefore, the study also verified the association of crop growth with the mosquito vector population and JE epidemiology. Moreover, Liyi County is part of a major cotton production area and JE epidemic county in Shanxi Province [30, 31]. The present article not only provided the first information to our knowledge on the potential causal factors of JE cases in a non-rice-growing area of northern China but also suggested that irrigation management in cotton fields could be as important for JE control in this region as rice irrigation management in southern China.

In this study, the pig number per pigsty was positively related to the vector density in the villages (VVD) and the vector infection index in the pigsty (PVI). Pigs are major hosts of *Cx. tritaeniorhynchus* and are a key reserve host of the JE virus [32], and the close relationship among the pig numbers and vector density [33] and vector infection rate [33], and the number of human JE cases [34] has been documented in many studies. Generally, the pig is the preferred host of *Cx. tritaeniorhynchus* [35], so it would be expected that the density of this mosquito in pigsties would be positively correlated with that of pigs. In this study, there was a positive connection between the pig numbers and vector density together with the vector infection index, confirming the importance of pigs in the JE transmission process. Our study site is located in the northern dry-farming region of China where no rice is grown; thus, our results may suggest that pig farming plays a key role in the incidence of JE in this region. Regarding the key risk of JE infection in pigs, special management considerations should be applied to decrease the infection rate among local people in Liyi County. The management choices used to reduce the incidence of JE in northern China could include the pigsty location, placing mosquito control devices in pigsties, immunizing pigs against JE, and JE virus surveillance in pigs.

In addition to the geo-environmental features (e.g., the growing crop and pig), the geographic features displayed a relation to the vector density. The gully, which originated from soil erosion caused by seasonal floods, is a typical geographic feature of the Loess Plateau in the northern part of China [36]. In general, the gully could flood in summer and be dry in winter [36]. Thus, floods in gullies could be viewed as a major water source in summer when mosquito vectors are prevalent in local areas. In this study, the vector density in the pigsty was negatively correlated with the distance of the pigsty from the gully. Although no previous study has confirmed the relationship between the PVD and the gully, the dependence between the PVD and gully here would be reasonable if the gully was considered a water flood in summer. As shown by other studies, the distance from the rice field was negative relative to the JE vector density in the rice growing area [37], and the JE vector density was also negatively dependent on the distance from the water flood in southern China [38]. In this study, our results hinted that seasonally flooding gullies could be the key geo-environmental feature affecting the local JE vector population. Therefore, the gully should be considered an important breeding source for vector mosquitoes to be investigated, and the corresponding management measures should be applied to the gully to decrease the vector density by slowing down the gully erosion or eliminating the gully.

The road length was negatively related to the vector density in the pigsties here. In this study, the road was located in a 1-km buffer area around the pigsty; thus, the road length was the same as the road density in a 1-km buffer area around the pigsty. Generally, the density of roads ranked as high risk for the spread of West Nile Virus (WNV) in Mississippi, USA [39]; moreover, the road net also affects the spatial distribution of dengue vector mosquito species [40] and malaria risk [41]. However, the present investigation seemed to show the opposite effect of road in the above references setting, in which the negative association between the PVD and the road length implied that the higher the road density was, the lower the vector density in the local area. Why? The environmental and socioeconomic background in the local region provided answers for this result. In the study area, the higher road density around the pigsties indicated that the site was closer to downtown areas where there were fewer pigs and fewer cotton areas around the pigsty because local people depended primarily on vegetables and orchards for their economic interests. In addition, a closer downtown area indicates a higher living status and sanitary rank for the local people, which could also decrease the breeding sites for mosquito vectors. Overall, the negative relation of the vector density to road density was consistent with the ecoepidemiology of JE and its vector.

In this study, the geographic features, geo-environmental features, vector density, vector infection index, and JE case number comprised the GEVJ [geography-environment-vector (density and infection index)-JE (cases)] intercorrelation net in the study area. The number of JE cases is influenced by the vector density, vector infection index, human-vector interaction rate, and level of immunization [42–44]. In addition, the vector density, vector infection index, and number of JE cases are the key aspects of the JE transmission cycle, and the spatial distribution of these three parameters effectively characterizes the local JE epidemiology [45, 46]. In this study, the mosquito vector density, vector infection index, and JE cases were interrelated and determined by the geo-environmental features through the correlated net in the study area. This study not only demonstrated that there was an association among the vector density, vector infection index, and JE cases, as in previous studies, but it also shed light on the relationship between the geo-environmental features and JE epidemiological factors, including the vector density, vector infection index, and JE cases.

Apparently, model 1.3 and model 2.1 did not add new knowledge because the vector infection indices (PVII and VVII) depended respectively on the vector density (PVD and VVD) by the definition of VI. However, these two models confirm the vector density influence on the vector infection and quantitated the relationship between VII and VD, which could aid the control of JE epidemiology. Moreover, this article constructed an interrelated net covering the JE epidemiological factors and geo-environmental features, which would assist local disease control staff in decreasing the JE transmission risk in the study area. For example, the local residents could relocate pigsties to decrease the vector density according to the spatial associations of vector density with the geo-environmental features, which would result in a lower vector density, vector infection index, and human infection cases successively.

## Conclusion

This study was the first quantitative analysis on the association among geo-environmental features, vector density, vector infection index, and incidence of JE in northern China, which could help understand and control the epidemiology of JE in northern China. First, environmental management, e.g., cotton irrigation management as well as slowing down or eliminating the gully, could be applied to decrease mosquito vector density. Second, selecting pigsty location, placing mosquito control devices in pigsties, immunizing pigs against JE, and JE virus surveillance in pigs should also be used, considering the key role of pig. Finally, regression models among various geo-environmental factors, the vector density, vector infection index, and number of JE cases provided a useful predictive tool to aid JE epidemiology control in the study area.

## Supplementary Information

**Additional file 1: Table S1.** Environmental factors potentially correlated with the abundance of mosquito vectors of Japanese B encephalitis in pigsties in Liyi County, Shanxi, China. **Table S2.** Comparison of OLS and spatial model estimates of PVD in Liyi County, Shanxi, China. **Table S3.** Distribution test and spatial autocorrelative test on the vector density, infection index and JE cases. **Figure S1.** Moran’s scatter plot (**a**) and envelope slopes (**b**) for PVD in Liyi County, Shanxi, China. **Figure S2.** Moran’s scatter plot (**a**) and envelope slopes (**b**) for PVII in Liyi County, Shanxi, China. **Figure S3.** Moran’s scatter plot (**a**) and envelope slopes (**b**) for VVD in Liyi County, Shanxi, China. **Figure S4.** Moran’s scatter plot (**a**) and envelope slopes (**b**) for VVII in Liyi County, Shanxi, China. **Figure S5.** Moran’s scatter plot (**a**) and envelope slopes (**b**) for VJC in Liyi County, Shanxi, China. **Model S0.1.**–**Model S0.7.** models for regression analysis of PVD and selected environmental factors. **Model S1.1.**–**Model S1.3.** Models for generalized linear model (GLM) regression analysis of PVII. **Model S2.1.**–**Model S2.5.** models generalized linear regression on the vector infection index and geo-environmental factors. **Model 3.1.**–**Model S3.4.** Models for generalized linear regression on village JE cases and geo-environmental factors.

## Data Availability

The datasets used and/or analyzed during the current study are available from the corresponding author on reasonable request.

## Abbreviations

JE: Japanese encephalitis
ETM: Enhanced thematic mapper
GIS: Geographic information systems
RS: Remote sensing
GPS: Global positioning systems
GEVJ: Geo-environment-vector-JE
CDC: Centers for Disease Control
RT-PCR: Reverse transcription polymerase chain reactions
PVD: Pigsty vector density
PVII: Pigsty vector infection index
VVD: Village vector density
VVII: Village vector infection index
VJC: Village Japanese encephalitis case number
GLM: Generalized linear model
OLS: Ordinary least squares
AIC: Akaike info criterion
LM: Lagrange multiplier
ANOVA: Analysis of variance
WNV: West Nile virus
