# Supplementary material for: Spatial statistical and environmental correlation analyses on vector density, vector infection index and Japanese encephalitis cases at the village and pigsty levels in Liyi County, Shanxi Province, China
Source: Parasit Vectors. 2022 May 19;15:171. doi: 10.1186/s13071-022-05305-8 (PMC9118647; doi:10.1186/s13071-022-05305-8)
Supplement: Supplementary file 1 — Additional file 1: Table S1. Environmental factors potentially correlated with the abundance of mosquito vectors of Japanese B encephalitis in pigsties in Liyi County, Shanxi, China. Table S2. Comparison of OLS and spatial model estimates of PVD in Liyi County, Shanxi, China. Table S3. Distribution test and spatial autocorrelative test on the vector density, infection index and JE cases. Figure S1. Moran’s scatter plot (a) and envelope slopes (b) for PVD in Liyi County, Shanxi, China. Figure S2. Moran’s scatter plot (a) and envelope slopes (b) for PVII in Liyi County, Shanxi, China. Figure S3. Moran’s scatter plot (a) and envelope slopes (b) for VVD in Liyi County, Shanxi, China. Figure S4. Moran’s scatter plot (a) and envelope slopes (b) for VVII in Liyi County, Shanxi, China. Figure S5. Moran’s scatter plot (a) and envelope slopes (b) for VJC in Liyi County, Shanxi, China. Model S0.1.–Model S0.7. models for regression analysis of PVD and selected environmental factors. Model S1.1.–Model S1.3. Models for generalized linear model (GLM) regression analysis of PVII. Model S2.1.–Model S2.5. models generalized linear regression on the vector infection index and geo-environmental factors. Model 3.1.–Model S3.4. Models for generalized linear regression on village JE cases and geo-environmental factors. [file 13071_2022_5305_MOESM1_ESM.docx]

**Table1** Environmental factors potentially correlated with the abundance of mosquito vectors of Japanese B Encephalitis in pigsties in Liyi County, Shanxi, China

| Variable name | Definition |
| --- | --- |
| Polygon factors | |
| A_COTTON | Area of the cotton within a 1 KM buffer around village(pigsties) |
| A_CORN | Area of the corn within a 1 KM buffer around village(pigsties) |
| A_orchard | Area of the orchard within a 1 KM buffer around village(pigsties) |
| A_VEGETAB | Area of the vegetable within a 1 KM buffer around village(pigsties) |
| A_WHEAT | Area of the wheat within a 1 KM buffer around village(pigsties) |
| Line factors | |
| L_RIVER | Length of river within a 1 KM buffer around village(pigsties) |
| L_ROAD | Length of district road within a 1 KM buffer around village(pigsties) |
| Distance | |
| D_GULLY | Distance between village(pigsties) and gullies |
| D_RAOD | Distance between village(pigsties) and district roads |
| D_RIVER | Distance between village(pigsties) and rivers |
| D_XANGJIE | Distance from village(pigsties) to the township boundary |
| D_YR | Distance from village(pigsties) to the Yellow River |
| Others | |
| PN | Number of adult pigs in the villages’ pigsties or individual pigsties |

**Table 2** Comparison of OLS and spatial model estimates of PVD in Liyi County, Shanxi, China

|  | OLS  Model | Spatial Lag Model | Spatial Error Model |
| --- | --- | --- | --- |
| Prob | 0.087 (F-statistic) | 0.011 (Robust LM (lag) test) | 0.009 (Robust LM (error) test) |
| Akaike Info Criterion | 331.381 | 333.269 | 331.181 |

**Table 3** Distribution test and spatial autocorrelative test on the vector density, infection index and JE cases

|  | | From the Normal Distribution | | From the Poisson Distribution | | |
| --- | --- | --- | --- | --- | --- | --- |
|  |  | PVD | VVD | PVII | VVII | VJC |
| Distribution test | Kolmogorov-Smirnov Z. | 1.179 | 0.526 | 1.169 | 0.150 | 0.115 |
|  | Asymp. Sig. (2-tailed) | 0.124 | 0.945 | 0.130 | 1.000 | 1.000 |
| Spatial autocorrelative test | Moran’s I | 0.381 | 0.08 | -0.077, | -0.21 | -0.015 |
|  | pseudo-P-value | 0.027 | 0.238 | 0.547 | 0.084 | 0.567 |

**Fig. 1** Moran’s scatter plot (a) and envelope slopes (b) for PVD in Liyi County, Shanxi, China. The Moran’s scatter line (blue lines) lies out of envelope slopes (red lines) indicated there was spatial autocorrelative of PVD. Abbreviations: PVD, Pigsty Vector Density; W_PVD, spatial lag of PVD.

b

a

**
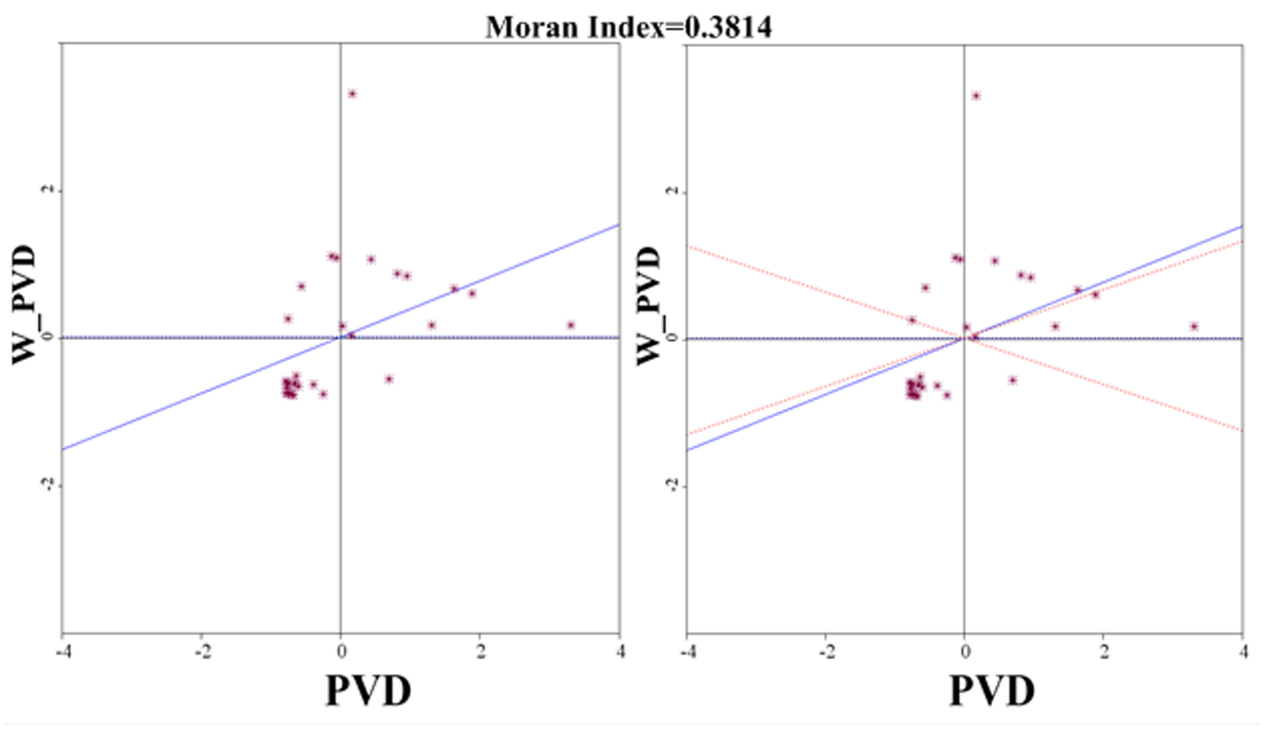
**

**Fig. 2** Moran’s scatter plot (a) and envelope slopes (b) for PVII in Liyi County, Shanxi, China. The Moran’s scatter line (blue lines) lies inside of envelope slopes (red lines) indicated there was non-spatial autocorrelative of PVII. Abbreviations: PVII, Pigsty Vector Infection Index; W_PVII, spatial lag of PVII.


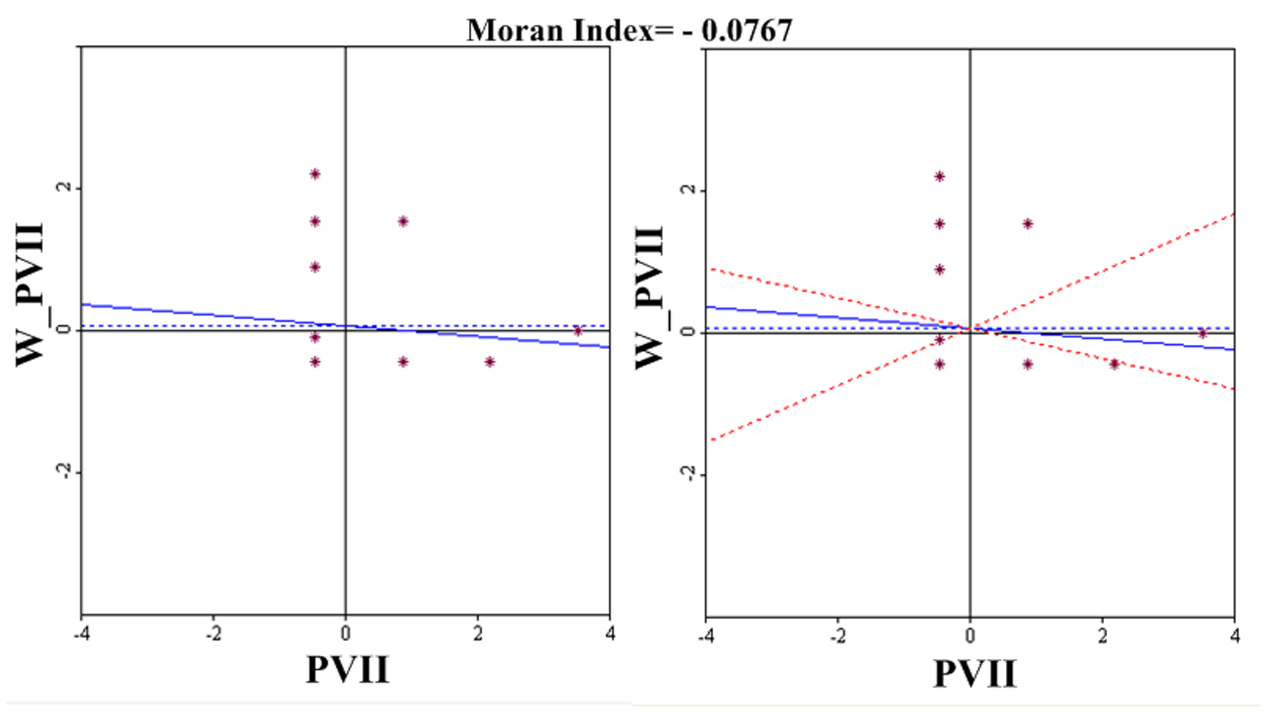


b

a

**Fig. 3** Moran’s scatter plot (a) and envelope slopes (b) for VVD in Liyi County, Shanxi, China. The Moran’s scatter line (blue lines) lies inside of envelope slopes (red lines) indicated there was non-spatial autocorrelative of VVD. Abbreviations: VVD, Village Vector Density; W_VVD, spatial lag of VVD.

a

b

**
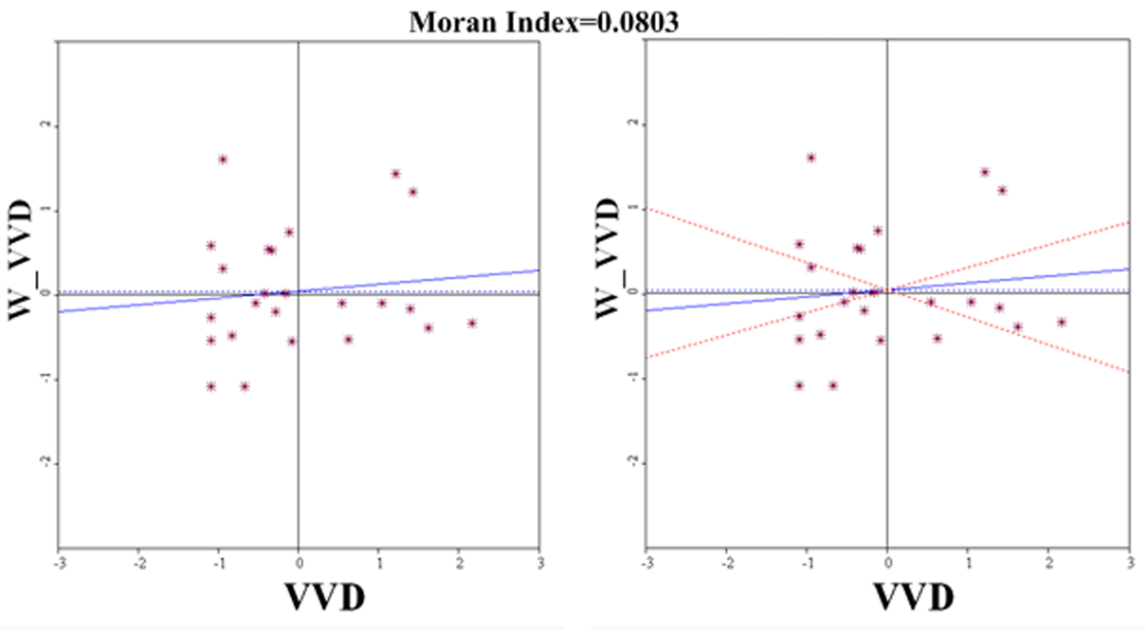
**

**Fig. 4** Moran’s scatter plot (a) and envelope slopes (b) for VVII in Liyi County, Shanxi, China. The Moran’s scatter line (blue lines) lies inside of envelope slopes (red lines) indicated there was non-spatial autocorrelative of VVII. Abbreviations: VVII, Village Vector Infection Index; W_VVII, spatial lag of VVII.


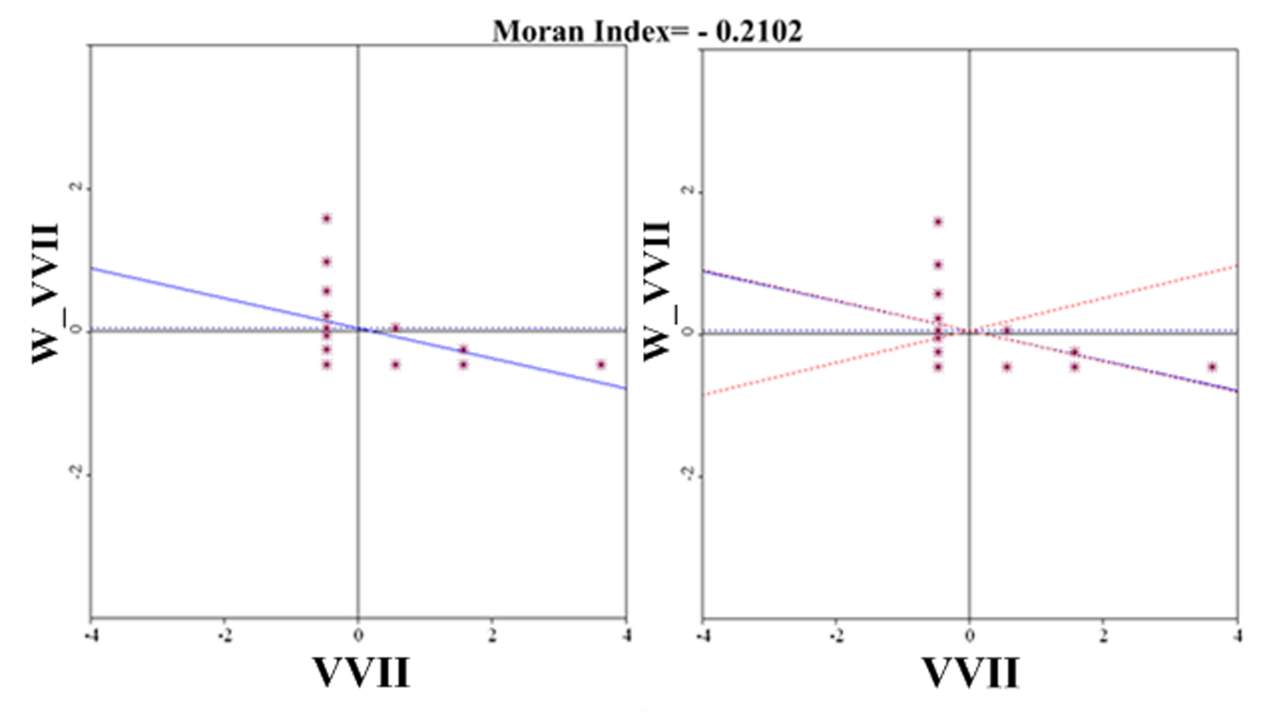


b

a

**Fig. 5** Moran’s scatter plot (a) and envelope slopes (b) for VJC in Liyi County, Shanxi, China. The Moran’s scatter line (blue lines) lies inside of envelope slopes (red lines) indicated there was non-spatial autocorrelative of VJC. Abbreviations: VJC, Village Japanese encephalitis Case number; W_VJC, spatial lag of VJC. **
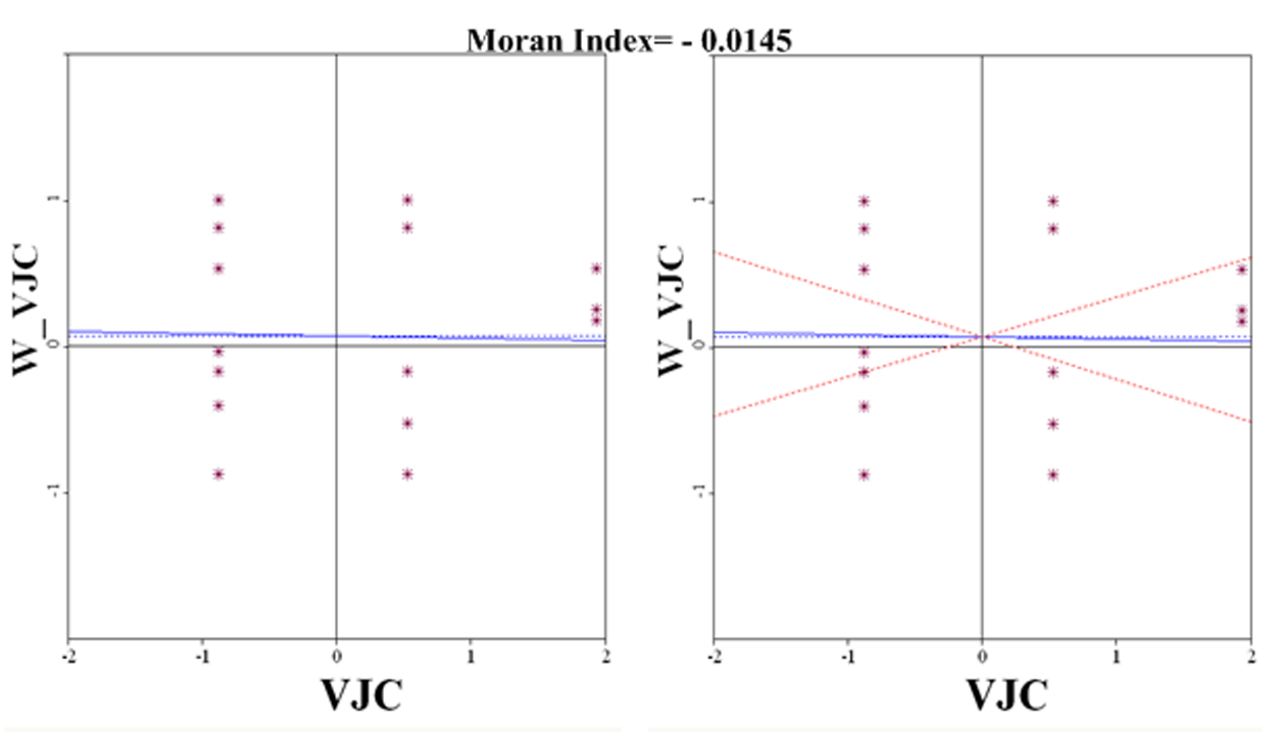
**

b

a

**Model-0.1～model-0.7** models for regression analysis of PVD and selected environmental factors

model-0.1: PVD= Intercept + b1(Cotton area) + ε, AIC value=330.309;

model-0.2: PVD= Intercept + b1(Gully Distance) + ε, AIC value=332.902;

model-0.3: PVD = Intercept + b1(Road Length) + ε, AIC value=327.502;

model-0.4: PVD = Intercept + b1(Cotton area) + b2(Gully Distance) + ε, AIC value=330.506;

model-0.5: PVD = Intercept + b1(Cotton area) + b2(Road Length) + ε, AIC value=327.341;

model-0.6: PVD = Intercept + b1(Gully Distance) + b2(Road Length) + ε, AIC value=330.297;

model-0.7: PVD = Intercept + b1(Gully Distance) + b2(Road Length) + b3(Cotton area) + ε, AIC value=328.275;

where the ε was the spatial error term.

**Model-1.1～model-1.3** Models for generalized linear model (GLM) regression analysis of PVII

model-1.1: Log link function (PVII) = Intercept + b1(PVD), AIC value=60.509;

model-1.2: Log link function (PVII) = Intercept + b1(Pig Number), AIC value=62.412;

model-1.3: Log link function (PVII) = Intercept + b1(PVD) + b2(Pig Number), AIC value=55.585.

**Model-2.1～model-2.5** Models for generalized linear regression on the vector infection index and geoenvironmental factors

model-2.1: Log link function (VVII) = Intercept + b1(VVD), AIC value=20.995;

model-2.2: Log link function (VVII) = Intercept + b1(VVD) + b2(River Length), AIC value=22.995;

model-2.3: Log link function (VVII) = Intercept + b1(Pig Number), AIC value=29.392

model-2.4: Log link function (VVII) = Intercept + b1(Pig Number) +b2(River Length), AIC value=27.045;

model-2.5: Log link function (VVII) = Intercept + b1(River Length), AIC value=29.238.

**Note that**：The pig number and VVD could not be input into the model at the same time because of the significant coefficient between the VVD and the pig number.

**Model-3.1～model-3.4** Models for generalized linear regression on village JE cases and geoenvironmental factors

model-3.1: Log link function (VJC) = Intercept + b1(VVII), AIC value=46.423;

model-3.2: Log link function (VJC) = Intercept + b1(VVII) + b2(Wheat area), AIC value=42.631;

model-3.3: Log link function (VJC)=Intercept + b1(VVII) + b2(Wheat area) +b3(Xiangjie Distance), AIC value=44.569;

model-3.4: Log link function (VJC)=Intercept+b1(VVII)+b2(Wheat area) +b3(Xiangjie Distance) +b4(Pig Number), AIC value=45.833.
